# Impact of offending vessel location on lateral spread response variations in hemifacial spasm patients

**DOI:** 10.3389/fneur.2025.1561134

**Published:** 2025-03-25

**Authors:** Feiyu Ding, Pan Li, Xiaozhou Zuo, Yong Xiao, Dong Wang, Yong Liu, Yuanjie Zou

**Affiliations:** ^1^Department of Neurosurgery, Nanjing BenQ Medical Center, The Affiliated BenQ Hospital of Nanjing Medical University, Nanjing, Jiangsu Province, China; ^2^Department of Neurosurgery, Affiliated Nanjing Brain Hospital, Nanjing Medical University, Nanjing, China

**Keywords:** hemifacial spasm, microvascular decompression, electrophysiological monitoring, lateral spread response, abnormal muscle response

## Abstract

**Objective:**

This study aims to investigate the impact of the offending vessel’s compression location on intraoperative lateral spread response (LSR) waveform parameters during microvascular decompression (MVD) for hemifacial spasm (HFS). Additionally, the study evaluates the clinical significance of LSR variations in intraoperative electrophysiological monitoring.

**Methods:**

A retrospective analysis was conducted on 72 patients with HFS who underwent MVD at Nanjing Brain Hospital between September 2021 and September 2023. Patients were categorized into two groups based on the compression site of the offending vessel on the facial nerve: the transitional zone (TZ) group and the attached segment (AS) group. General clinical characteristics, intraoperative LSR parameters, and postoperative outcomes were compared between groups. Statistical analyses focused on LSR latency, amplitude, and duration, as well as the patterns of LSR disappearance and postoperative complications.

**Results:**

The TZ group comprised 31 patients, while the AS group included 41. No significant differences were observed in baseline characteristics between groups. Intraoperative monitoring revealed that LSR disappearance was more frequently incomplete in the TZ group (11.1%) than in the AS group (*p* < 0.05). LSR latency was significantly longer in the AS group (*p* < 0.001), while the amplitude in the orbicularis oculi muscle was lower in the TZ group (*p* < 0.001). Additionally, LSR duration (T2) in the orbicularis oris (*p* < 0.05) and mentalis muscles (*p* < 0.01) was longer in the AS group, though the amplitude differences were not statistically significant. Postoperative outcomes showed no significant difference in effectiveness between the groups (AS: 92.7% vs. TZ: 93.5%, *p* = 0.882). Complications, such as facial palsy and hoarseness, were slightly more common in the AS group, whereas hearing loss and ataxia were more frequent in the TZ group. However, none of these differences reached statistical significance.

**Conclusion:**

The compression location of the offending vessel significantly influences LSR parameters, with longer latency and prolonged duration observed in the AS group. Despite these variations, postoperative outcomes and complications were comparable between groups. These findings highlight the importance of considering the compression location during MVD and the potential value of LSR monitoring in guiding surgical decision-making.

## 1 Introduction

Hemifacial spasm (HFS) is characterized by unilateral eyelid twitching associated with involuntary contractions of the facial muscles. It typically begins with intermittent spasms of the orbicularis oculi muscle, gradually spreading to involve the muscles of the face and the corners of the mouth (1). In severe cases, patients may experience an inability to open the eye, accompanied by tonic spasms of the facial muscles and deviation of the mouth (2). Triggers for these spasms include emotional stress, fatigue, and encounters with unfamiliar people. The precise pathogenesis of HFS remains unclear, but the prevailing hypothesis is based on the neurovascular compression (NVC) theory proposed by Gardner. This theory suggests that compression of the facial nerve root exit zone (REZ) by the offending vessel leads to demyelination of the nerve (3–5).

**Figure 1:**
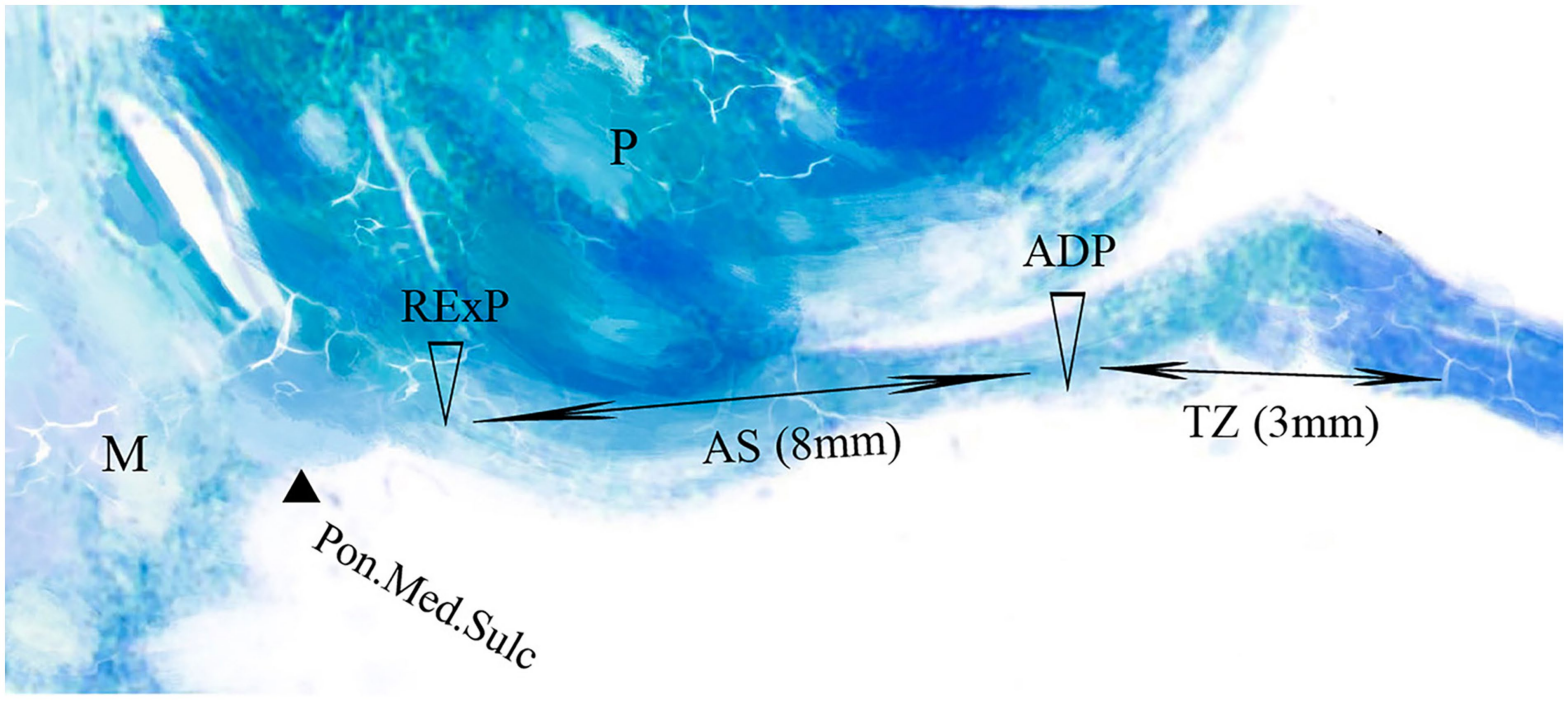
Segmental anatomy of the facial nerve. The figure illustrates the anatomical segments of the facial nerve. M denotes the medulla, P represents the pons, and Pon.Med.Sulc refers to the pontomedullary sulcus. The AS (attached segment) and TZ (transitional Zone) are highlighted. RExP indicates the root origin point, while ADP denotes the root departure point.

Jannetta pioneered microvascular decompression (MVD) surgery, which involves using the retrosigmoid approach to relieve the vessel from the facial nerve REZ under a microscope, achieving significant clinical success in treating HFS (6, 7). MVD is predominantly focused on the REZ region of the facial nerve. Tomii et al. (8) subdivided the facial nerve REZ into four parts based on the distribution of central myelin within the nerve (Figure 1): the root exit point (RExP), the attachment segment (AS), the root detachment point (RDP), and the transitional zone (TZ). Literature reports indicate that the AS segment is approximately 8–10 mm, with the TZ segment located 1–3 mm distal to the AS. Different compression points necessitate varying exposure techniques for NVC, posing challenges for the surgeon. In such cases, electrophysiological monitoring plays a crucial role in identifying the responsible vessel (9–12).

Intraoperative electrophysiological monitoring has been widely adopted in MVD surgeries for HFS across multiple centers. Lateral spread response (LSR), also known as abnormal muscle response (AMR), is the most commonly used monitoring potential (13, 14). The principle behind LSR involves stimulating one branch of the facial nerve and detecting abnormal muscle responses in muscles innervated by other branches. The prevailing theory suggests that intraoperative LSR aids in identifying the culprit vessel and assists in determining the optimal timing for surgical intervention (15, 16). Most studies on LSR focus on its relationship with surgical outcomes, with few reports addressing the significance of LSR parameters during surgery. Moreover, LSR exhibits various patterns of change intraoperatively, depending on the surgeon’s manipulations, which can be challenging. Therefore, this study aims to summarize the intraoperative LSR patterns and explore the relationship between LSR parameters and the location of the offending vessel (17–19).

## 2 Methods

A retrospective analysis was conducted on cases of primary hemifacial spasm (HFS) treated at Nanjing Brain Hospital between September 2021 and September 2023. The inclusion criteria were patients diagnosed with primary HFS who underwent microvascular decompression (MVD) of the facial nerve, had a follow-up period of at least 1 year, and were aged 18 years or older.

The diagnosis of HFS was confirmed through clear clinical symptoms, preoperative imaging that demonstrated a close relationship between the facial nerve and surrounding vessels, and a positive lateral spread response (LSR) on preoperative electrophysiological monitoring. The two most commonly used MRI sequences for the imaging diagnosis of hemifacial spasm are 3D-TOF-MRA (three-dimensional time-of-flight magnetic resonance angiography) and 3D-FIESTA (three-dimensional fast imaging employing steady-state acquisition). The 3D-TOF-MRA sequence provides high-resolution images of blood vessels, helping to identify vascular compression around the facial nerve, while the 3D-FIESTA sequence offers clear visualization of the relationship between the facial nerve and surrounding vessels, providing important imaging evidence for the diagnosis of hemifacial spasm.

Patients who had secondary HFS, failed to complete follow-up, or had severe underlying conditions such as psychiatric disorders or malignant tumors that could affect surgical outcomes were excluded from the study. Additionally, patients with a history of destructive treatments, including facial nerve resection or nerve ablation, prior to surgery were also excluded. Patients who received Botox injections within 6 months prior to surgery were excluded to avoid potential confounding effects on LSR parameters.

### 2.1 Grouping method

Preoperative imaging, including 3D-TOF-MRA and 3D-FIESTA sequences, was reviewed to assess the distance between the compressive vessel and the pontomedullary sulcus. The location of the compressive vessel relative to the facial nerve root exit zone was used as the basis for categorizing patients into two groups. In the AS group, the compression point was located within 8 mm of the root exit point (RExP), corresponding to the attachment segment of the facial nerve to the brainstem. In the TZ group, the compression point was situated 8–12 mm from the anterior-dorsal point (ADP), representing the transitional zone between the central and peripheral myelin. To ensure diagnostic consistency, imaging results were jointly evaluated by experienced neurosurgeons and radiologists. Additionally, intraoperative surgical videos were reviewed to assist in determining the exact location of the compression. This method facilitated a consistent and precise categorization of patients, enhancing the reliability of the results (Figure 1).

### 2.2 Observation indicators

Preoperative symptoms were classified into five grades according to Cohen’s classification (Table 1). Grade 0 indicates the absence of spasms or twitching. Grade I involves mild, brief eyelid tremors. Grade II is characterized by mild tremors of the eyelid and facial muscles, with a symmetrical facial appearance at rest. Grade III includes frequent, more pronounced spasms of the eyelid and facial muscles, leading to facial asymmetry at rest. Grade IV represents continuous spasms of the eyelid and face, with severe facial distortion during episodes, rendering the patient unable to perform daily tasks such as reading, driving, crossing the street, or cooking. In this grade, the nasolabial fold on the affected side becomes shallower, and the corner of the mouth droops.

**Table 1:** Preoperative severity of hemifacial spasm according to Cohen’s classification.

Classification	Clinical manifestation
0	No facial spasms or twitching
I	Mild eyelid fluttering with a short duration
II	Mild twitching of eyelid and facial muscles, with facial symmetry at rest
III	Frequent and pronounced twitching of eyelid and facial muscles, with facial asymmetry at rest
IV	Persistent facial spasms, severe facial distortion during episodes, inability to read, drive, cross the street, or cook; with a deepening of the nasolabial fold and drooping of the mouth corner on the affected side

Postoperative outcomes were categorized into four levels (Table 2): an “Excellent” outcome indicates complete resolution of spasms, a “Good” outcome denotes occasional mild twitching, a “Fair” outcome involves persistent mild spasms, and a “Poor” outcome signifies no improvement in symptoms following surgery.

**Table 2:** Postoperative assessment of HFS-MVD.

Outcome	Description
Excellent	Complete resolution of spasms
Good	Occasional mild twitching
Fair	Persistent mild twitching
Poor	No improvement in symptoms

### 2.3 Intraoperative electrophysiological monitoring method

All patients underwent intraoperative electrophysiological monitoring using the cascade system (USA). Surface electrodes were placed on the orbicularis oculi, orbicularis oris, and mentalis muscles to record facial electromyography (EMG) and lateral spread response (LSR). Monitoring was performed continuously from the initiation of anesthesia until the closure of the dura mater.

Stimulating needle electrodes were inserted subcutaneously at the zygomatic and mandibular branches of the facial nerve, with a pulse width of 0.3 milliseconds and an intensity ranging from 5 to 25 mA. The zygomatic branch stimulation electrode was positioned at the midpoint of the line connecting the lateral canthus and the external auditory canal on the affected side. The mandibular branch stimulation electrode was inserted under the skin at the angle of the mandible. Stimulation of the zygomatic branch recorded LSR in the orbicularis oris and mentalis muscles, while stimulation of the mandibular branch recorded LSR in the orbicularis oculi muscle.

To minimize the impact of muscle relaxants on LSR appearance, the anesthesiologist was instructed to discontinue or reduce muscle relaxants to the lowest effective dose before making the scalp incision. Facial nerve stimulation was performed at multiple time points, with each stimulation repeated five times or more: before the discontinuation of muscle relaxants, after anesthesia induction and before craniotomy, before dural opening, during cerebrospinal fluid release, before decompression, after decompression, and after dural closure. The criterion for a positive LSR was the appearance of a stable waveform with consistent latency across five consecutive stimulations.

### 2.4 Measurement method for LSR-related parameters

**Figure 2:**
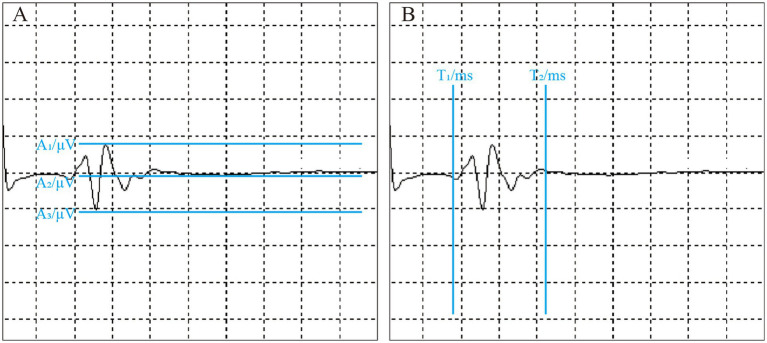
Intraoperative LSR parameter measurement. The figure shows the measurement of LSR parameters during surgery. All parameters are automatically labeled by the monitoring equipment. **(A)** Illustrates LSR amplitude-related parameters: A1 represents the peak, A2 denotes the baseline, and A3 indicates the trough. **(B)** Displays LSR latency-related parameters: T1 is the latency, and T2 is the time to return to baseline.

If a stable waveform is observed in five consecutive stimulations, the system automatically records the related parameters (Figure 2). T1 represents the time when the waveform begins, indicating the latency of the LSR. T2 denotes the duration of the waveform. A1 refers to the peak amplitude of the LSR, A2 is the baseline, and A3 corresponds to the trough of the waveform.

### 2.5 Surgical procedure

The patient was positioned in the park-bench position with the Mayfield head holder securing the head. The head was flexed forward and positioned 10° below the horizontal. A classic Jannetta approach was used for microvascular decompression (MVD), involving a posterior fossa craniotomy via a suboccipital retrosigmoid approach. A 5–7 cm incision was made just behind the ear, along the hairline, followed by a layered dissection through the skin, exposing the lateral aspect of the occipital bone and the mastoid surface. The exposure extended 1.5 cm above the occipital protuberance and down to the digastric groove.

A bone window of approximately 3 × 3 cm was created using a drill, revealing the angle between the sigmoid and transverse sinuses. Under the microscope, the dura mater was incised in a circular fashion and suspended. The arachnoid membrane over the cerebellomedullary cistern was gently dissected, and cerebrospinal fluid (CSF) was slowly released. After satisfactory cerebellar retraction, the cerebellar hemisphere was gently retracted to free the arachnoid membrane in the cerebellopontine angle (CPA) cistern. Further dissection was performed to expose the facial and acoustic nerve complex.

The Teflon was carefully placed between the offending vessel and the nerve, adjusting the padding until the LSR waveform disappeared satisfactorily. The surgical field was irrigated with saline to ensure no bleeding. The dura mater was watertight closed, and a titanium plate was secured to the skull. The muscle layers and scalp were then sutured in layers.

### 2.6 LSR variability patterns

**Figure 3:**
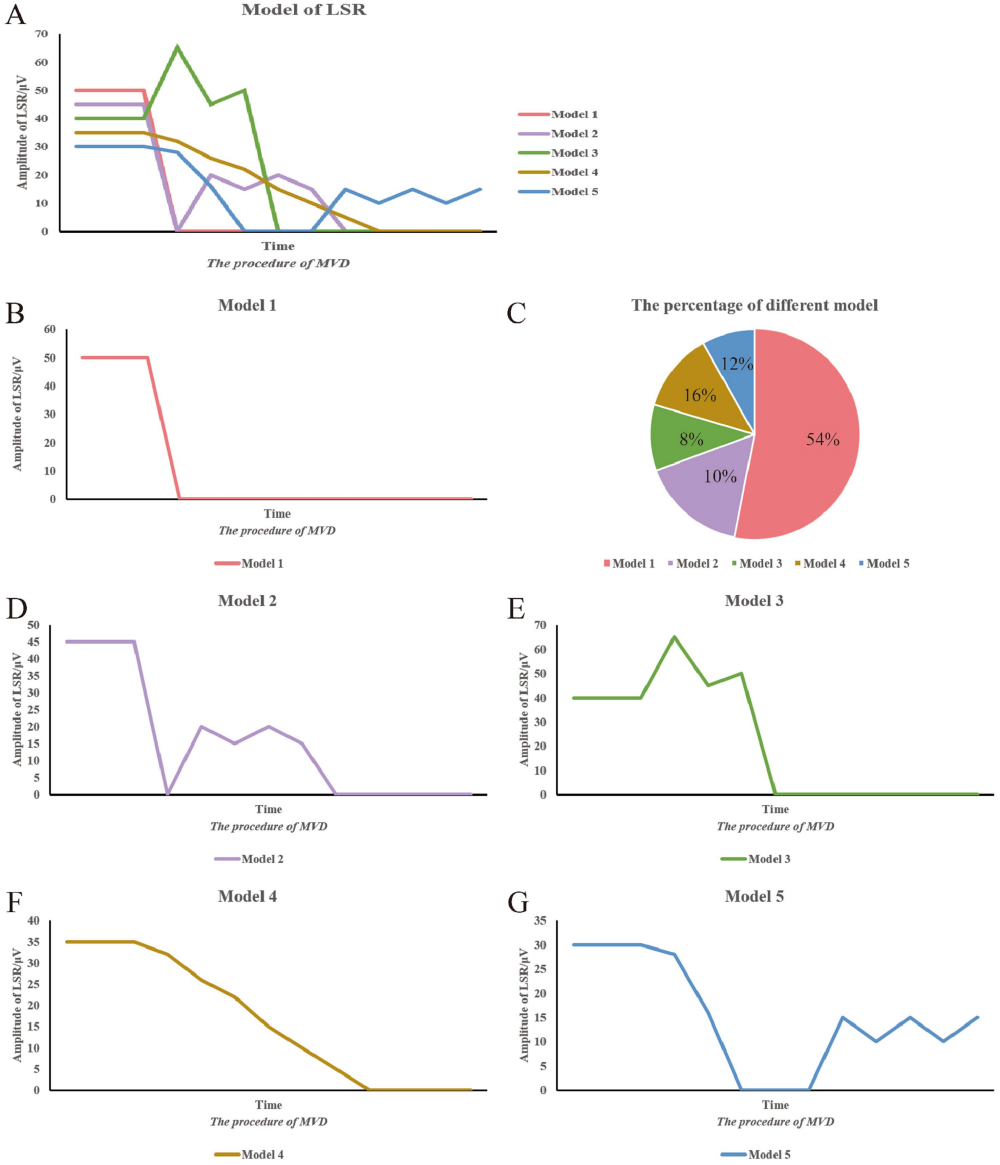
Summary of LSR patterns during surgery. This figure summarizes the various LSR patterns observed during surgery. The vertical axis represents the peak of the LSR waveform, while the horizontal axis represents the progression of the surgical procedure. **(A)** Different model of LSR. **(B)** Model 1. **(C)** The percentage of different model. **(D)** Model 2. **(E)** Model 3. **(F)** Model 4. **(G)** Model 5.

**Figure 4:**
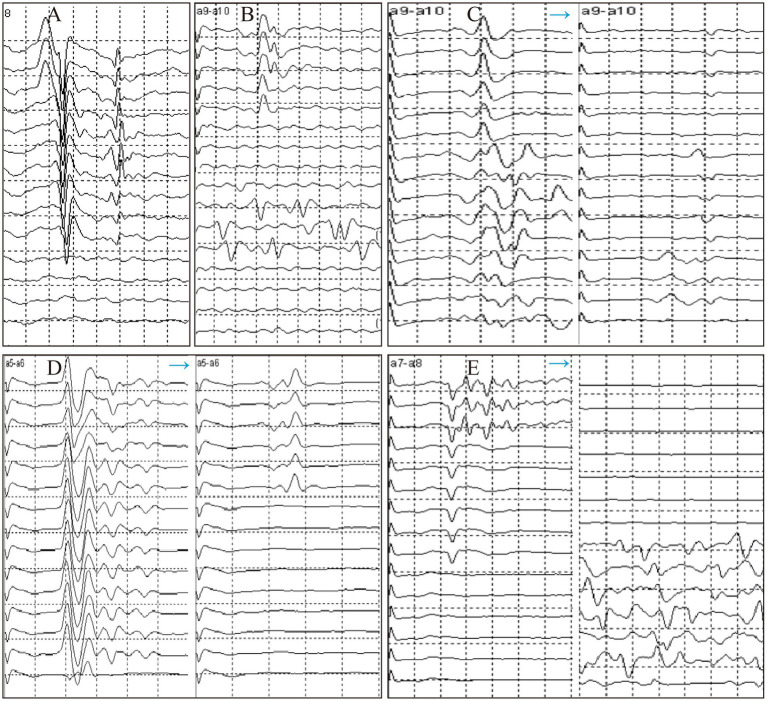
Trends of LSR disappearance patterns during surgery. **(A)** Model 1. **(B)** Model 2. **(C)** Model 3. **(D)** Model 4. **(E)** Model 5.

The variability patterns of the LSR were analyzed based on changes in the peak of the waveform throughout the surgical procedure, and curve graphs were plotted (Figures 3, 4). In Pattern 1, the LSR completely disappeared satisfactorily after placing the Teflon, and was not reproducible. Pattern 2 involved the waveform reappearing after the Teflon was placed, but eventually disappearing as the surgeon adjusted its position. Pattern 3 showed an increase in the peak amplitude of the LSR after Teflon was inserted, which then eventually disappeared following adjustments to its position. Pattern 4 described a gradual disappearance of the peak while adjusting the Teflon. Pattern 5 involved the waveform disappearing and then reappearing, but ultimately failing to disappear satisfactorily.

### 2.7 Data analysis

Statistical analysis was performed using commercial software (SPSS version 26.0, IBM Corp., Armonk, NY, United States). Continuous variables were expressed as mean ± standard deviation. Statistical tests used for comparisons between groups included the t-test for continuous variables and the chi-square test for categorical data. Differences between groups were assessed using an independent samples *t*-test. A *p*-value of less than 0.05 was considered statistically significant. Graphs were generated using GraphPad Prism 9 and Origin 2021 software.

## 3 Results

### 3.1 Baseline characteristics

This study included a total of 72 patients with HFS, with 41 patients in the AS group and 31 in the TZ group. The baseline characteristics of the patients are summarized in Table 3. The mean age of onset was 54.51 years in the AS group, which was slightly higher than the 52.41 years observed in the TZ group. There were 38 cases of left-sided onset and 34 cases of right-sided onset. Preoperative Cohen grading revealed 10 patients at grade II, 42 at grade III, and 20 at grade IV. In terms of the types of offending vessels, the most common was the posterior inferior cerebellar artery (PICA) with 30 cases, followed by the anterior inferior cerebellar artery (AICA) with 25 cases. Dual vessel compression (e.g., vertebral artery + AICA, vertebral artery + PICA, AICA + PICA) was observed in 17 cases. The number of facial nerve indentations was higher in the AS group (19 cases) compared to the TZ group (11 cases). There were no statistically significant differences in the baseline characteristics between the two groups (Table 3).

**Table 3:** General information.

Group	AS group	TZ group	Total	Statistic	*p*-value
Cases	41	31	72		
Gender				0.101^a^	0.751
Male	18 (56.3%)	14 (43.8%)	32		
Female	21 (52.5%)	19 (47.5%)	40		
Age (year)	54.51 ± 9.60	52.41 ± 11.02		0.859^b^	0.393
Side				0.039^a^	0.844
Left	21 (55.3%)	17 (44.7%)	38		
Right	18 (52.9%)	16 (47.1%)	34		
Cohen score				0.981^a^	0.612
II	4 (40%)	6 (60%)	10		
III	23 (54.8%)	19 (45.2%)	42		
IV	9 (45%)	11 (55%)	20		
Culprit vessel				4.615^a^	0.329
AICA	10 (40%)	15 (60%)	25		
PICA	14 (46.7%)	16 (53.3%)	30		
VA + AICA	2 (40%)	3 (60%)	5		
VA + PICA	4 (66.7%)	2 (33.3%)	6		
AICA+PICA	5 (83.3%)	1 (16.7%)	6		
Indentations				0.856^a^	0.355
Yes	19 (63.3%)	11 (36.7%)	30		
No	22 (52.4%)	20 (47.6%)	42		

a Denotes the *χ*^2^ value.

b Represents the *t* value.

### 3.2 Distribution of LSR disappearance patterns

Table 4 and Figure 3C present the distribution of the five LSR disappearance patterns. Pattern 1 was observed in 39 cases (54.2%), with 30 cases in the AS group and 9 in the TZ group. Pattern 2 occurred in 7 cases (10%), with 3 cases in the AS group and 4 in the TZ group. Pattern 3 was noted in 6 cases (8%), with 5 cases in the AS group and 1 in the TZ group. Pattern 4 appeared in 12 cases (16%), with 4 cases in the AS group and 8 in the TZ group. Pattern 5 was observed in 8 cases (12%), with 3 cases in the AS group and 5 in the TZ group. Statistical analysis revealed significant differences between the groups (*p* < 0.05).

**Table 4:** LSR disappearance pattern statistics.

Group	AS group	TZ group	Total	Statistic	*p*-value
Total	45 (62.5%)	27 (37.5%)	72	11.859^a^	0.013
Pattern 1	30 (76.9%)	9 (23.1%)	39		
Pattern 2	3 (42.9%)	4 (57.1%)	7		
Pattern 3	5 (83.3%)	1 (16.7%)	6		
Pattern 4	4 (33.3%)	8 (66.7%)	12		
Pattern 5	3 (37.5%)	5 (62.5%)	8		

a Denotes the *χ*^2^ value.

### 3.3 LSR parameters

**Figure 5:**
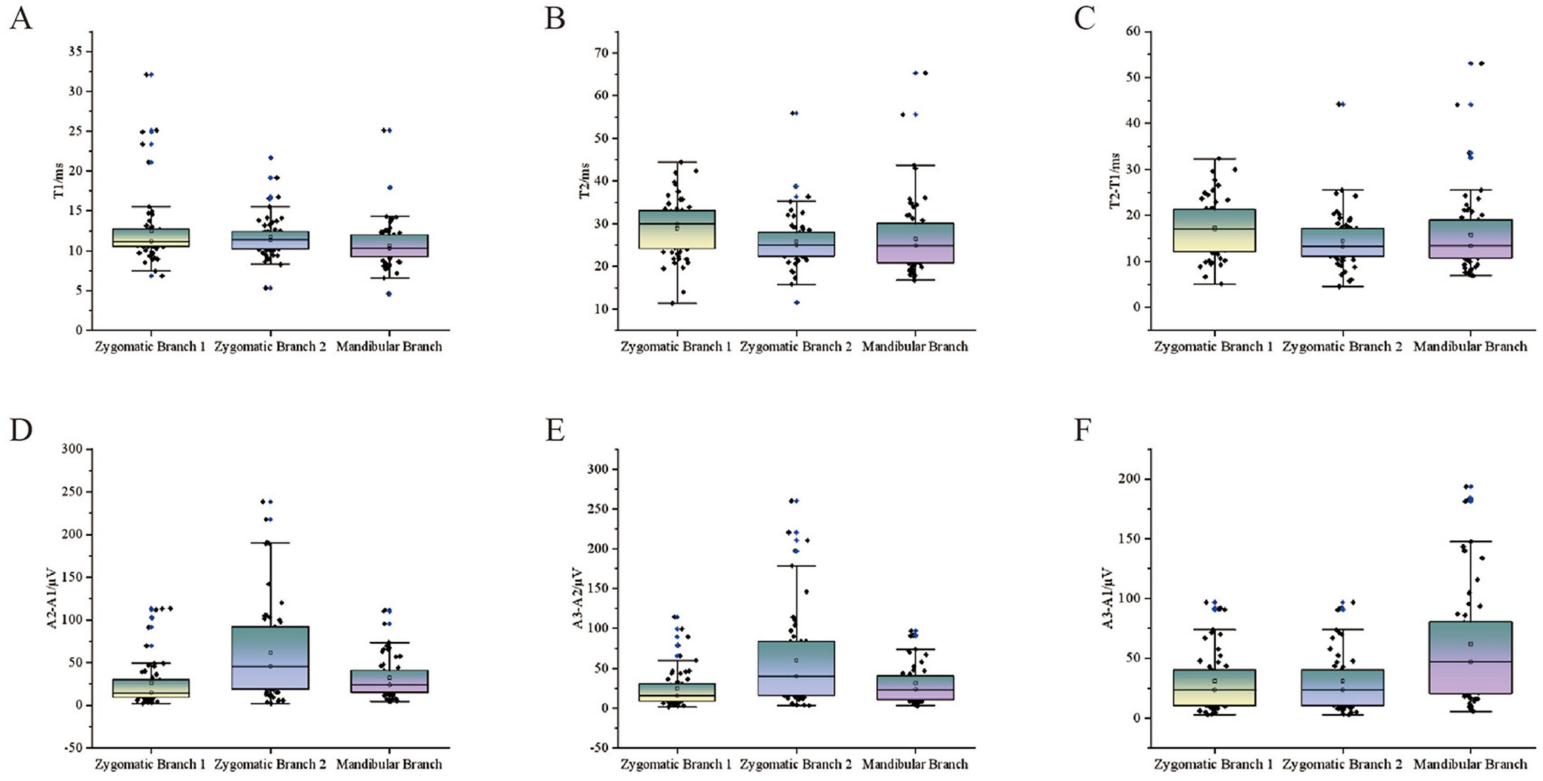
Scatter plots of LSR parameters for all patients. The scatter plots show the relationship between LSR parameters and stimulation sites for all patients. The vertical axis represents various LSR-related monitoring parameters, while the horizontal axis includes different stimulation sites: stimulation 1 (orbicularis oris), stimulation 2 (mentalis), and mandibular branch stimulation (orbicularis oculi). **(A)** T1. **(B)** T2. **(C)** T2-T1. **(D)** A2-A1.**(E)** A3-A2. **(F)** A3-A1.

Figure 5 illustrates the distribution of LSR-related parameters. Overall, T1 ranged from 4.6 ms to 32.08 ms, with a median of 11.17 ms. T2 ranged from 11.32 ms to 65.29 ms. The difference between T2 − T1 ranged from 4.57 ms to 53.07 ms. The amplitude difference between A2 − A1 ranged from 2.05 μV to 238.38 μV, while the difference between A3 − A2 ranged from 1.49 μV to 260.05 μV.

**Figure 6:**
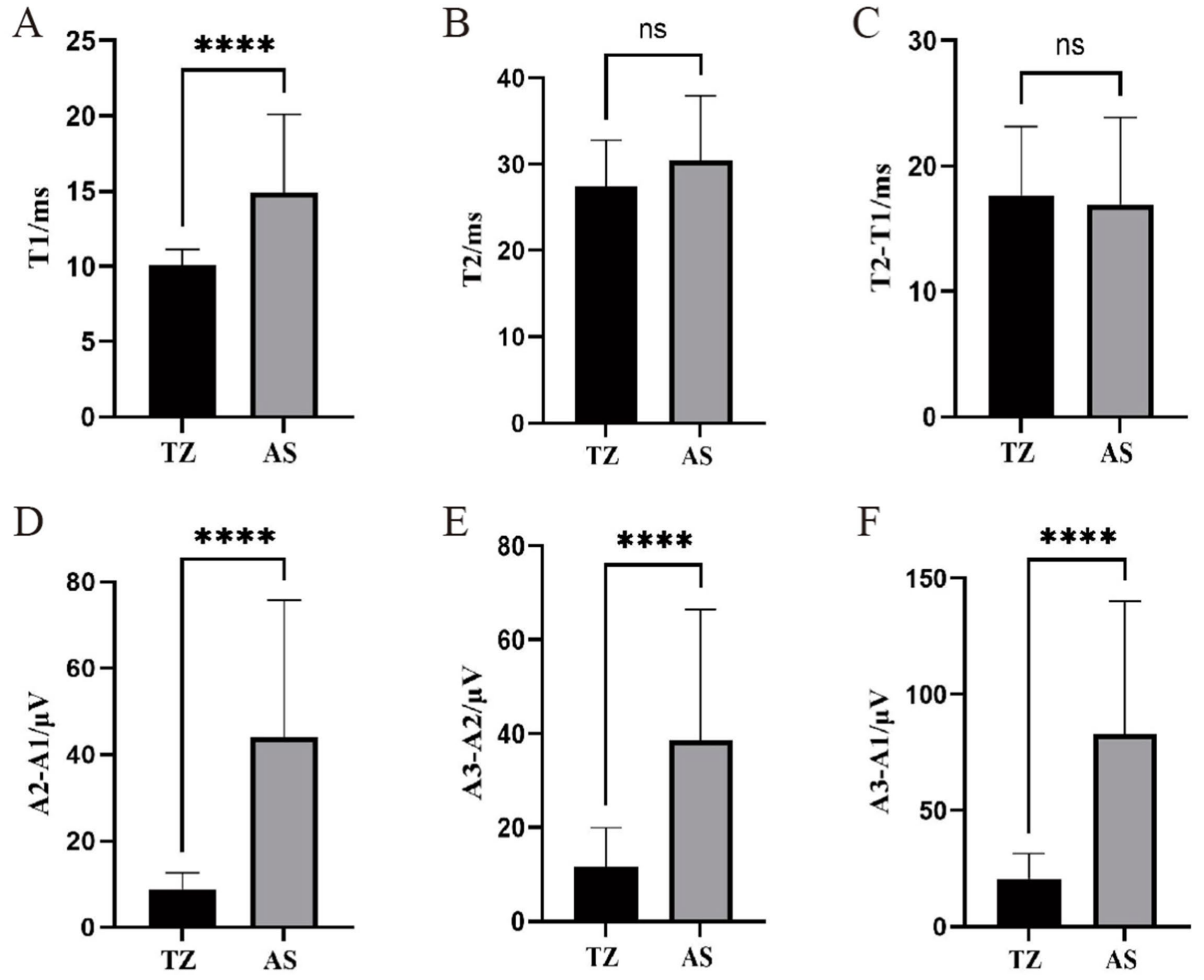
Comparison of LSR parameters in orbicularis oculi muscle between two groups. ^****^p < 0.001 (statistically significant difference). ns, not significant. This figure compares LSR parameters between the two groups for the orbicularis oculi muscle. T1: Time of waveform onset, indicating LSR latency. T2: Duration of the waveform. A1: Peak amplitude of LSR. A2: Baseline amplitude. A3: Trough amplitude. **(A)** T1. **(B)** T2. **(C)** T2-T1. **(D)** A2-A1.**(E)** A3-A2. **(F)** A3-A1.

For the orbicularis oculi muscle LSR, T1 (latency) was significantly longer in the AS group compared to the TZ group, with a *p*-value of <0.001. T1 ranged from 6.83 ms to 32.08 ms, while T2 ranged from 11.32 ms to 44.38 ms. No significant difference was found between the groups for T2 and the difference between T2 − T1. The amplitude differences A2 − A1 and A3 − A2 ranged from 2.05 μV to 113.37 μV and 1.49 μV to 114.33 μV, respectively. The difference between A3 − A1 ranged from 3.54 μV to 211.18 μV. Differences in the amplitude-related parameters for the orbicularis oculi muscle LSR were statistically significant (*p* < 0.001) (Figure 6).

**Figure 7:**
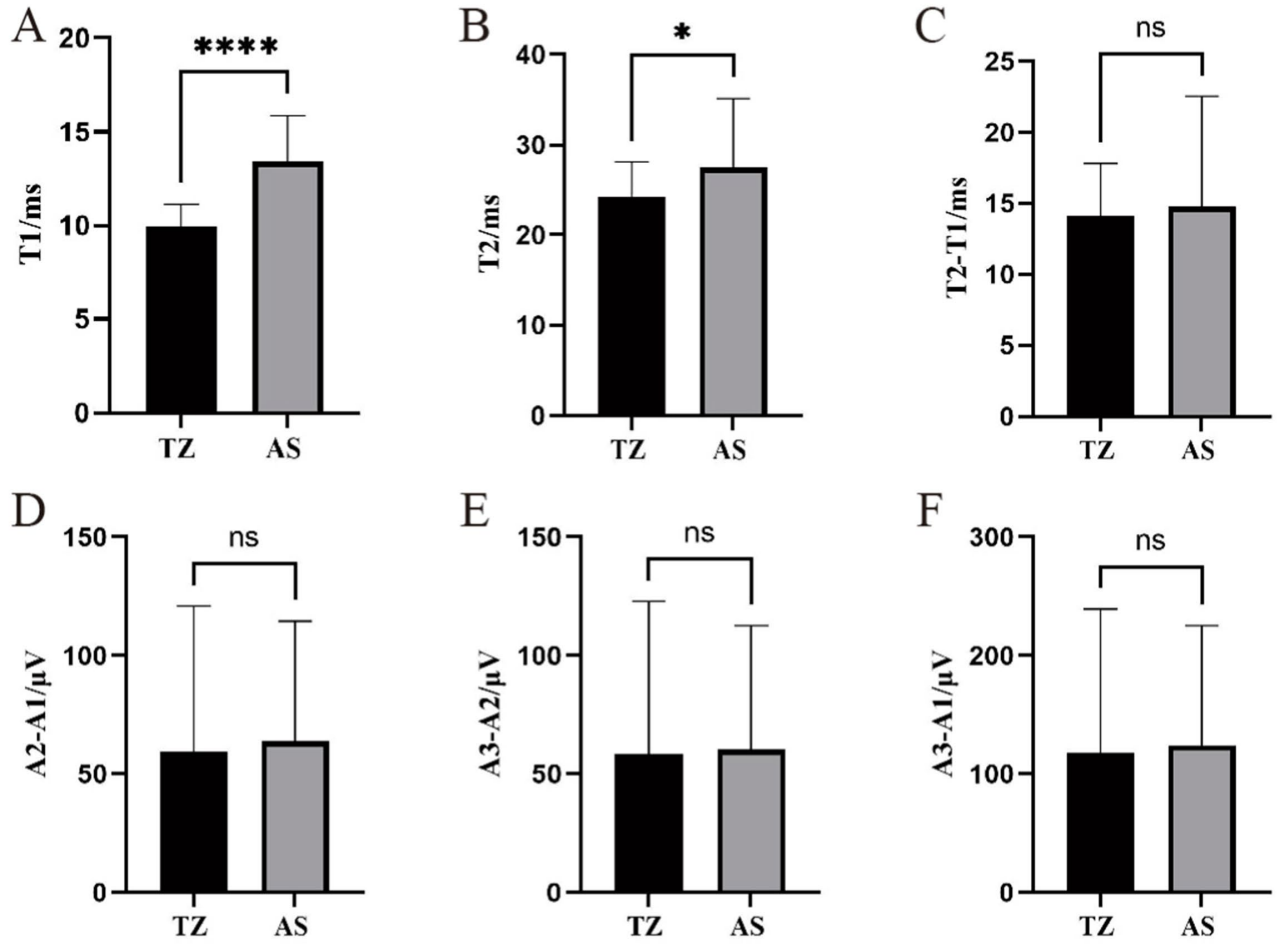
Comparison of LSR parameters in the orbicularis oris muscle between two groups. ^****^Statistically significant difference between the two groups (*p* < 0.001). ^*^Statistically significant difference between the two groups (*p* < 0.05). ns: No statistically significant difference between the two groups. This figure displays the comparison of LSR parameters between the two groups for the orbicularis oris muscle. **(A)** T1. **(B)** T2. **(C)** T2-T1. **(D)** A2-A1.**(E)** A3-A2. **(F)** A3-A1.

For the orbicularis oris muscle LSR, the latency in the AS group was significantly longer than in the TZ group, with a *p*-value of <0.001. The latency ranged from 11.51 ms to 55.89 ms. T2 also ranged from 11.51 ms to 55.89 ms, with a significant difference between the groups (*p* < 0.05). The difference between T2 − T1 ranged from 4.57 ms to 44.21 ms, with no significant difference between groups. The amplitude differences A2 − A1, A3 − A2, and A3 − A1 ranged from 2.13 μV to 238.38 μV, 3.35 μV to 260.05 μV, and 5.97 μV to 458.88 μV, respectively. No significant differences were observed between the groups for the amplitude-related parameters (Figure 7).

**Figure 8:**
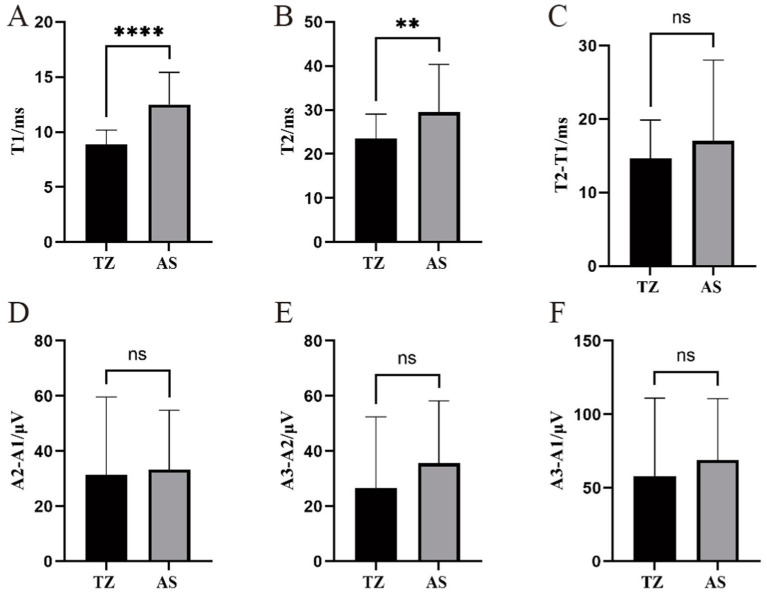
Comparison of LSR parameters in the mentalis muscle between two groups. ^****^Statistically significant difference between the two groups (*p* < 0.001). ^**^Statistically significant difference between the two groups (*p* < 0.01). ns: No statistically significant difference between the two groups. This figure compares LSR parameters between the two groups for the mentalis muscle. **(A)** T1. **(B)** T2. **(C)** T2-T1. **(D)** A2-A1.**(E)** A3-A2. **(F)** A3-A1.

The latency of LSR recorded from the mentalis muscle primarily ranged from 4.6 ms to 25.11 ms, with significantly longer latencies observed in the AS group compared to the TZ group (*p* < 0.001). T2 ranged from 16.73 ms to 65.29 ms, with a statistically significant difference between the groups (*p* < 0.05). The difference between T2 − T1 ranged from 6.94 ms to 53.07 ms, with no significant difference between the groups. The amplitude differences A2 − A1, A3 − A2, and A3 − A1 ranged from 4.46 μV to 111.77 μV, 2.86 μV to 96.87 μV, and 7.93 μV to 208.64 μV, respectively. No significant differences in the amplitude-related parameters were observed between the groups (Figure 8).

### 3.4 Surgical outcomes

In this study, up to 96% of patients with HFS experienced relief from symptoms after undergoing HFS-MVD, with no signs of residual symptoms during follow-up. Table 5 details the postoperative outcomes and complications for the cohort.

**Table 5:** Postoperative data.

Group	AS group	TZ group	Total	Statistic	*p*-value
Outcome				0.022^a^	0.882
Effective	38 (55.1%)	31 (44.9%)	69		
Ineffective	1 (33.3%)	2 (66.7%)	3		
Complications				2.102^a^	0.835
Facial palsy	3 (60%)	2 (40%)	5		
Hoarseness	2 (66.7%)	1 (33.3%)	3		
Drinking cough	0 (0.0%)	1 (100%)	1		
Hearing loss	2 (40%)	3 (60%)	5		
Intracranial infection	2 (40%)	3 (60%)	5		
Ataxia	1 (33.3%)	2 (66.7%)	3		

a Denotes *χ*^2^ value.

**Figure 9:**
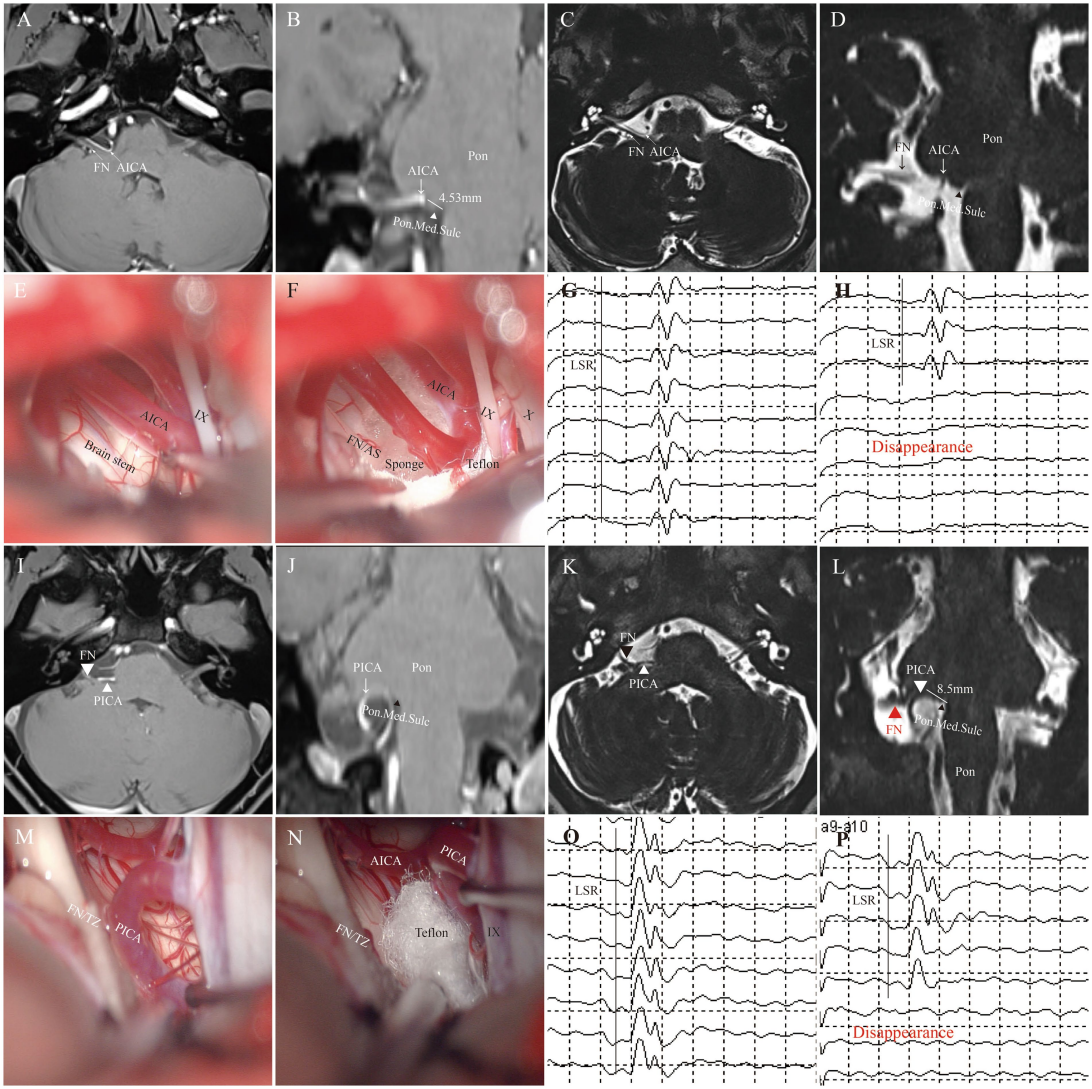
Representative cases. This figure illustrates typical cases of nerve compression points (AS: **A–H**; TZ: **I–P**). **(A–D)** Preoperative imaging shows the compression point in the facial nerve attachment segment (approximately 4.53 mm from the pontomedullary junction). **(E,F)** Intraoperative assessment confirms that the compression point is in the AS segment. **(G,H)** Intraoperative LSR monitoring. **(I–L)** Preoperative imaging shows the compression point located in the TZ segment (approximately 8.5 mm from the nerve origin). **(M,N)** Intraoperative video confirms that the compression point is in the TZ segment. **(O,P)** Intraoperative electrophysiological monitoring shows satisfactory disappearance of LSR. (FN, facial nerve; AS, attached segment; TZ, transitional zone; AICA, anterior inferior cerebellar artery; PICA, posterior inferior cerebellar artery; Pon.Med.Sulc, pontomedullary sulcus).

The incidence of postoperative complications was lower in the AS group compared to the TZ group. Postoperative facial paralysis was observed in 3 cases in the AS group and 2 cases in the TZ group. Voice hoarseness was reported in 2 patients in the AS group and 1 patient in the TZ group. Postoperative aspiration during drinking was only seen in the TZ group. Intracranial infections occurred in 2 patients in the AS group and 3 in the TZ group. Ataxia was observed in 2 patients in the TZ group and only 1 in the AS group. No major postoperative complications such as intracranial hemorrhage or death were reported in this cohort. The differences in postoperative complications between the two groups were not statistically significant (see Figure 9).

The incidence of intracranial infections (5/72) in our cohort was primarily associated with minor CSF leaks or dural tears during craniotomy. These infections were promptly managed with antibiotic therapy, and no cases of meningitis or brain abscess were observed. Prolonged operative duration and the technical complexity of the retrosigmoid approach may have contributed to the infection risk. To address this, we recommend optimizing dural closure techniques, reducing operative time, and enhancing sterile protocols in future cases.

## 4 Discussion

This study investigated the impact of the location of the offending vessel on LSR parameters in patients undergoing MVD for hemifacial spasm HFS. Our findings highlight significant differences in LSR parameters and disappearance patterns based on the compression point location (AS vs. TZ), contributing to a deeper understanding of intraoperative electrophysiological monitoring in HFS surgery.

The lower rate of complete LSR disappearance in the TZ group may reflect anatomical differences in neural vulnerability. The TZ, located distal to the AS, contains transitional myelin that is more susceptible to chronic compression (8). This could delay electrophysiological recovery compared to the AS group, where decompression directly targets the root exit zone.

We observed that LSR latency, particularly T1, was significantly longer in the AS group compared to the TZ group for all recorded muscles (orbicularis oculi, orbicularis oris, and mentalis). This suggests that the compression at the AS delays neural conduction more than at the TZ. These results, to some extent, support the peripheral hypothesis of HFS pathogenesis (20). According to the peripheral hypothesis, the compression causes a localized disruption in the nerve’s ability to propagate electrical signals, potentially altering the function of the facial nerve and contributing to the development of HFS. This disruption is more pronounced when compression occurs at the AS, where there may be less myelinated fibers and more chronic mechanical stress, further delaying the conduction velocity.

Additionally, amplitude-related differences (A2 − A1, A3 − A2) were statistically significant for the orbicularis oculi muscle but not for the orbicularis oris or mentalis muscles. Furthermore, the distribution of LSR disappearance patterns varied significantly between the AS and TZ groups, with Pattern 1 being predominantly associated with the AS group and Pattern 4 with the TZ group.

The role of LSR as a monitoring tool during MVD has been extensively discussed (21–25), but few studies have explored the influence of the anatomical location of the offending vessel on LSR parameters. Previous research (13, 16, 26–28) has primarily focused on the disappearance of LSR as an indicator of adequate decompression, but our findings suggest that different compression points contribute to distinct electrophysiological signatures. This nuanced understanding aligns with existing evidence, where variations in neural conduction were attributed to anatomical differences in the facial nerve’s exposure to vascular compression. However, our study is among the first to quantitatively analyze these differences and their clinical implications.

The differences in LSR parameters can be hypothesized to stem from variations in neural conductivity based on the anatomical location of the compression. The TZ, being closer to the root exit zone (REZ), may allow for more efficient transmission of electrical signals due to its higher density of myelinated fibers. In contrast, the AS is associated with areas where neural fibers may be less myelinated or subjected to chronic compression, potentially leading to delayed conduction velocities and altered signal amplitudes. This hypothesis aligns with the known physiological properties of the facial nerve, where myelination significantly affects conduction speed and waveform stability. Future studies using histopathological analysis of compression sites could validate this hypothesis.

Our findings have significant implications for intraoperative decision-making during MVD. The distinct LSR disappearance patterns observed in the AS and TZ groups provide critical insight into tailoring surgical strategies. For instance, a prolonged T1 latency in the AS group may indicate the need for a more extensive decompression, while specific disappearance patterns (e.g., Pattern 4 in the TZ group) can serve as markers of adequate decompression. Additionally, understanding the typical range of LSR parameters for different compression sites may help surgeons predict postoperative outcomes and refine surgical techniques to minimize complications.

The observed LSR patterns provide critical insights that may enhance intraoperative decision-making during MVD. For instance, in cases with Model 1 (complete LSR disappearance after initial Teflon placement), surgeons may confidently conclude adequate decompression. Conversely, Model 4 (gradual LSR attenuation) may necessitate iterative adjustments of Teflon position. The longer LSR latency in the AS group implies that surgeons should allow sufficient time for electrophysiological stabilization before confirming decompression efficacy.

This study has several limitations. First, the sample size, while sufficient for detecting statistical differences, limits the generalizability of the findings. A larger cohort is needed to confirm these results and explore additional variables, such as the duration of symptom onset and severity of nerve compression. Second, the absence of histopathological data on the compression sites limits our ability to confirm the proposed mechanisms underlying LSR variations. Third, our study did not evaluate long-term postoperative outcomes, such as recurrence rates, in relation to LSR parameters, which could provide further insights into the prognostic value of these findings. Finally, the use of a single electrophysiological monitoring system (cascade) may restrict the applicability of our results to other systems.

Another limitation of our study is that we did not specifically account for potential amplitude differences across muscle groups. Such differences could arise from variations in muscle thickness, electrode placement, or other technical factors. To further reduce potential biases, subsequent research should incorporate baseline normalization and standardized recording protocols.

Future studies should aim to validate these findings in larger, multicenter cohorts. Incorporating advanced imaging techniques, such as diffusion tensor imaging (DTI), could provide insights into the structural integrity of the facial nerve at different compression sites. Additionally, longitudinal studies assessing the correlation between LSR parameters and long-term outcomes would enhance our understanding of their predictive value. Exploring alternative monitoring techniques, such as nerve conduction velocity tests, may also provide complementary data for intraoperative decision-making.

## Data Availability

The original contributions presented in the study are included in the article/supplementary material, further inquiries can be directed to the corresponding authors.
